# The Cano-eMLST Program: An Approach for the Calculation of Canonical Extended Multi-Locus Sequence Typing, Making Comparison of Genetic Differences Among Bunches of Bacterial Strains

**DOI:** 10.3390/microorganisms7040098

**Published:** 2019-04-03

**Authors:** Yen-Yi Liu, Ji-Wei Lin, Chih-Chieh Chen

**Affiliations:** 1 Central Regional Laboratory, Center for Diagnostics and Vaccine Development, Centers for Disease Control, Taichung 40855, Taiwan; current788@gmail.com; 2 Institute of Medical Science and Technology, National Sun Yat-sen University, Kaohsiung 80424, Taiwan; jwlin@imst.nsysu.edu.tw; 3 Rapid Screening Research Center for Toxicology and Biomedicine, National Sun Yat-sen University, Kaohsiung 80424, Taiwan; 4 General Institute of Clinical Medicine, Kaohsiung Medical University, Kaohsiung 80708, Taiwan

**Keywords:** molecular typing, next-generation sequencing (NGS), core-genome multi-locus sequence typing (cgMLST), feature-selection

## Abstract

Extended multi-locus sequence typing (eMLST) methods have become popular in the field of genomic epidemiology. Before eMLST methods can be applied in epidemiological investigations, the selection of a suitable scheme is critical. The core genome scheme (cgMLST) has become the most popular eMLST approach for strain typing in the epidemiological domain. In addition to strain typing, many public health researchers and clinical microbiologists wish to investigate which genes cause genetic differences between compared strains. Therefore, a tool that can be used to extract canonical genes with an eMLST scheme would be particularly useful. In this study, we present cano-eMLST, a well-designed program that applies a feature-selection methodology to create a canonical locus combination with discriminatory power by traversing a genetic relatedness tree based on a user-selected scheme. The cano-eMLST program is provided mainly to help infectious disease laboratory researchers identify potential factors related to bacterial pathogenesis. The core program (tree-traversing approach) of cano-eMLST is implemented in Perl and Python. All the necessary dependencies and environmental settings are provided in the encapsulated version (VirtualBox or VMware) and self-installation version (all use source code and libraries).

## 1. Introduction

Pulsed-field gel electrophoresis (PFGE) molecular typing technology has been successfully employed to investigate foodborne disease epidemics, particularly by the nationwide molecular classification of foodborne bacterial disease monitoring network (PulseNet), which was established in 1996 [1]. Currently, the disease surveillance network has expanded beyond the United States to a global foodborne disease surveillance network known as PulseNet International [2]. PFGE is a standard typing tool used in PulseNet laboratories. This typing method is highly reliable and reproducible, and the typing results can be easily interpreted. However, PFGE is laborious and time-consuming and must be undertaken by skilled technicians. Studies have demonstrated that PFGE cannot effectively type certain species such as *Shigella sonnei* and *Salmonella enterica* serovar Enteritidis [3,4]. Multisite variability repeat sequence analysis was once a promising alternative to PFGE, but the method is highly species specific and not a common bacterial population tool. Furthermore, a comparison of its subgroup profiles is difficult across different laboratories [5,6]. Therefore, the optimal typing method for bacteria is whole-genome sequencing (WGS). With the advancement of next-generation sequencing (NGS) technology, WGS is likely to supersede PFGE as the PulseNet standard typing method in the near future.

However, the NGS platform typically generates millions of short sequences, and the analysis of numerous WGS sequences to generate the required information (e.g., regarding genotyping and resistance to different strains) is a challenge. Most employees in conventional laboratories lack expertise in bioinformatics, and therefore a simple and easy-to-use analytical platform is required for automating the analysis of WGS primitive sequence fragments and for performing genotypic comparisons of different strains in the laboratory. Whole-genome multi-locus sequence typing (wgMLST) is a suitable method for decoding genes (gene fingerprints) specific to a particular strain on the basis of the bacterial genome and NGS data. If wgMLST profiles can be generated from a common genome in the pan-genome database and compared between laboratories, they can be used as the standard data type for WGS and in infectious disease surveillance networks such as PulseNet.

The wgMLST approach is becoming popular, and therefore many extended multi-locus sequence typing (eMLST) schemes, which involve different locus combinations within the whole-genome scheme, continue to be developed and evaluated by numerous public health research groups [7,8,9,10,11,12,13,14]. To use an eMLST method in bacterial molecular typing, the selection of a suitable typing scheme is critical. However, creating a scheme appropriate for use in the epidemiological typing of a specific bacterial species is challenging. In addition to the use of eMLST for typing, researchers usually wish to know the critical differences between the genomes they are comparing for antimicrobial and pathogenesis studies. Therefore, a user-friendly hands-on tool that helps to create a canonical typing scheme which is reduced to a human-readable quantity is crucial, particularly for infectious disease researchers seeking to identify potential factors related to bacterial pathogenesis. In this study, we present the program cano-eMLST, which uses a tree-traversing feature-selection approach to achieve this objective.

## 2. Materials and Methods

The genomes of bacterial strains are usually composed of core genomes in various proportions (genes present in all strains) and an accessory genome (genes not present in all strains) [15]. The cano-eMLST program (https://sourceforge.net/projects/cano-emlst/files/) operates according to user-selected bacterial genomes, through which it creates a database that recruits the core genes and all accessory genes of the included bacterial strains (pan-genome scheme). In the database created, each collected gene is assigned to a corresponding locus (gene group) as an allele according to the sequence identity. Next, the nonredundant alleles for each locus are serially numbered. The user-selected genomes are then compared with the created allele database using BLASTN [16], and the serial allele numbers (allelic profile) are taken from the schematic loci. The converted allelic profiles (one profile for one genome) can then be compared to generate a Hamming distance matrix (HDM). An unweighted pair group method with arithmetic mean (UPGMA) tree can then be constructed using the HDM. Our tree-traversing and feature-selection approaches can then be employed to walk through all the nodes of the dendrogram and select the most important loci that are distinguishable for each split simultaneously. After the tree traversal is complete, the final selected loci are defined for the highly discriminatory scheme.

To evaluate the schemes selected using the cano-eMLST approach, two empirical datasets consisting of 31 *Listeria monocytogenes* isolates and 10 *Escherichia coli* isolates were used [17]. The cano-eMLST program comprises five functional modules, namely the Contig Annotator, the pan-genome allele database (PGAdb) Builder, the pgMLST Profiler, the Dendro Plotter, and the Loci Extractor, which perform all the aforementioned steps. The Contig Annotator is used for annotating genome contigs, the PGAdb Builder is used for creating the pan-genome allele database (PGAdb) on the basis of user-selected genomes, the pgMLST Profiler is used for generating allelic profiles by comparing user-selected genomes against the adjustable scheme selected from the constructed PGAdb, the Dendro Plotter is utilized for depicting UPGMA cladograms by using the allelic profiles generated from the same PGAdb, and the Loci Extractor is used for extracting the most important loci. The detailed descriptions of these five modules of the cano-eMLST program are provided in the following sections.

### 2.1. Contig Annotator

**Figure 1:**
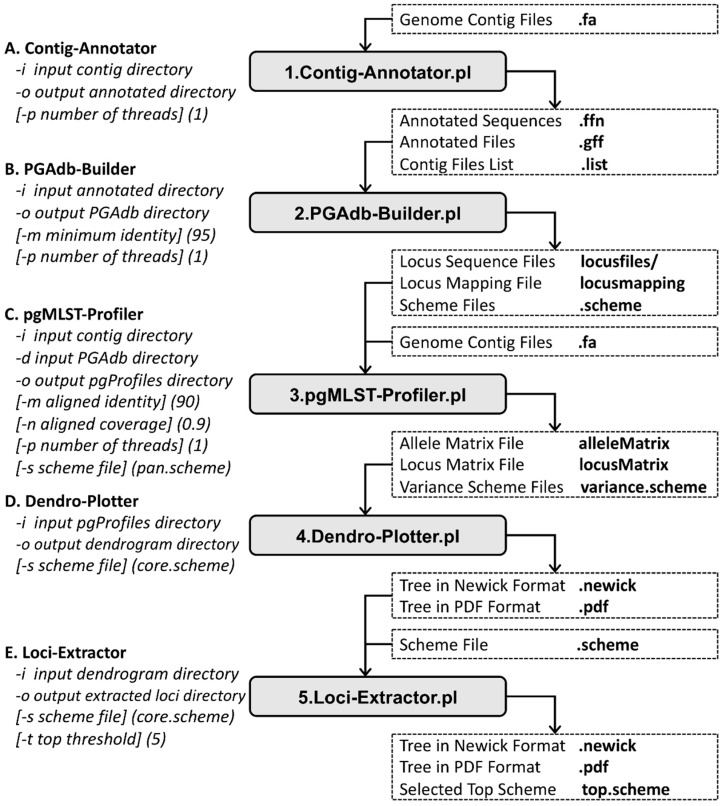
Schematic of cano-eMLST.

In the Contig Annotator step (Figure 1A), users import bacterial genome contigs (at least five genomes are required) in the FASTA format. The expensive computation required for contig annotation means that a computer with multicore processors for parallel processing is suggested for setting up the platform. In the platform, Prokka-v1.13 [18] is used for genome annotation.

### 2.2. PGAdb Builder

In the PGAdb Builder step (Figure 1B), users import annotated bacterial genome files (at least five genomes are required) in the .ffn and .gff formats. In the platform, Roary-v3.12.0 [19] is used for PGAdb construction. The running messages of the program appear in the terminal, and a log file is saved simultaneously.

### 2.3. pgMLST Profiler

In the pgMLST Profiler step (Figure 1C), users enter (1) the directory of bacterial genomes, (2) the directory of the PGAdb, (3) the output pgProfiles directory, and (4) the threshold of sequence identity and aligned coverage which create allelic profiles through blasting against the allele sequences within the selected scheme. The pan-genome scheme is selected by default if users do not specify a user-defined scheme file. All running messages appear in the log console, and the allelic profiles are generated locally.

### 2.4. Dendro Plotter

In the Dendro Plotter step (Figure 1D), users enter (1) the result directory of the pgMLST Profiler, (2) the output directory of the Dendro Plotter, and (3) the selected scheme for plotting the dendrogram. The core genome scheme is selected by default if users do not specify a user-defined scheme file. We employ the ETE3 Toolkit [20] for tree visualization. The dendrogram is plotted automatically and can be saved as a .pdf or .newick file.

### 2.5. Loci Extractor

In the Loci Extractor step (Figure 1E), users enter (1) the result directory of the Dendro Plotter, (2) the output directory of the Loci Extractor, (3) the selected scheme, and (4) the threshold for the selection of the most important loci for plotting the dendrogram. The pseudocodes of the tree-traversal and feature-extraction procedures are presented in Table 1. We employ the ETE3 Toolkit [20] for tree visualization. The dendrogram is plotted automatically and can be saved as a .pdf or .newick file.

## 3. Results

**Figure 2:**
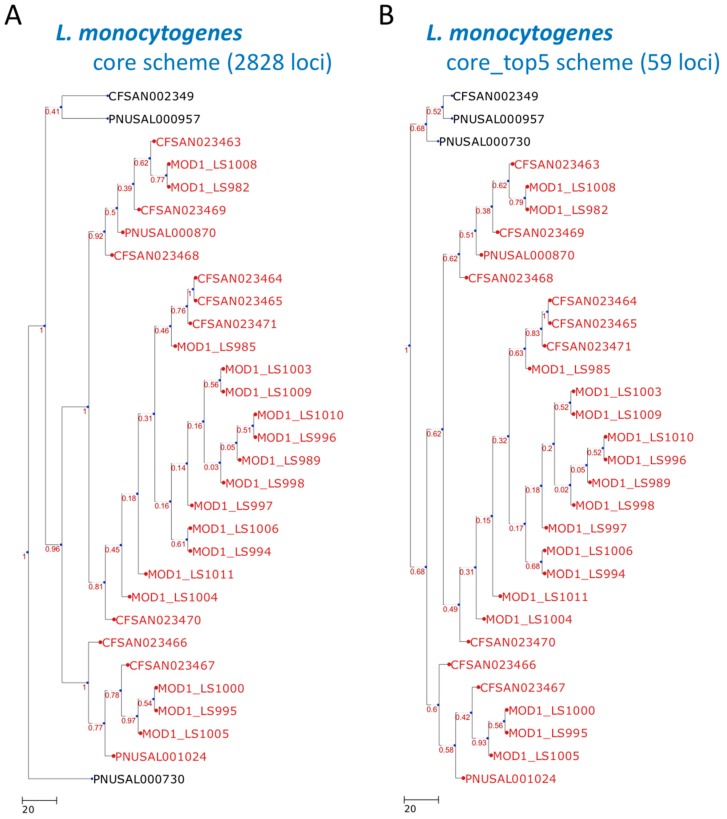
Dendrogram constructed with cgMLST profiles for 31 *Listeria monocytogenes* isolates. The cgMLST trees were generated according to the (**A**) core scheme (2828 loci) and (**B**) core_top5 scheme (59 loci). The outbreak or event-related taxa are colored red. The core scheme refers to the core genome scheme generated in step 2 by the PGAdb Builder. The core_top5 scheme is a subset of the core scheme that unites the five most discriminatory loci for each split. The scheme was generated in step five by the Loci Extractor.

**Figure 3:**
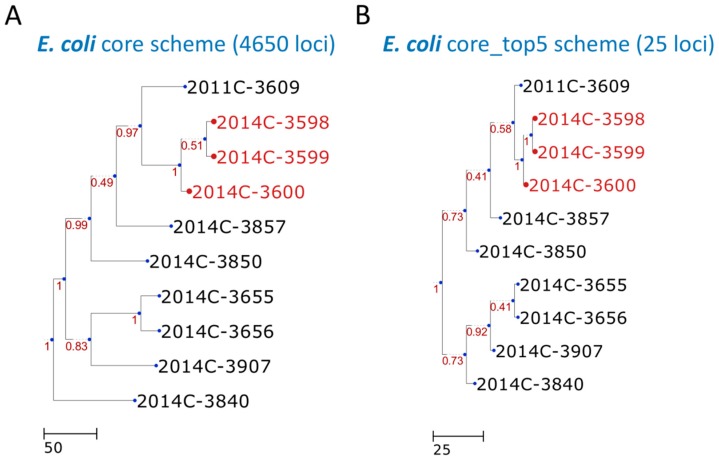
Dendrogram constructed with cgMLST profiles for ten *Escherichia coli* isolates. The cgMLST trees were generated according to the (**A**) core scheme (4650 loci) and (**B**) core_top5 scheme (25 loci). The outbreak or event-related taxa are colored red. The core scheme refers to the core genome scheme generated in step 2 by the PGAdb Builder. The core_top5 scheme is a subset of the core scheme that unites the five most discriminatory loci for each split. The scheme was generated in step five by the Loci Extractor.

We used two benchmark datasets comprising 28 empirical outbreak isolates and three outgroups belonging to *Listeria monocytogenes* and three empirical outbreak isolates and seven outgroups belonging to *Escherichia coli* [17] to demonstrate the use of the cano-eMLST program. These datasets were used to help measure the closeness of the predicted dendrogram (Figure 2B and Figure 3B) to the original dendrogram (Figure 2A and Figure 3A). Input files of genomic data used for cano-eMLST were required to be in the FASTA format. The time required by the Contig Annotator and PGAdb Builder steps to process data concerning the 31 isolates was approximately 1 h. The pgMLST Profiler and Dendro Plotter steps each took approximately 2 min to be completed for the data of the 31 isolates. The final step of the traversing-tree (31 isolates) required 5 min to complete. After cano-eMLST processing, the final selected loci were reduced to 59 for the *Listeria monocytogenes* set (Figure 2) and 25 for the *Escherichia coli* set (Figure 3). These results can be compared with the core genome scheme comprising 2828 and 4650 loci for the *Listeria monocytogenes* and *Escherichia coli* sets, respectively. The resulting dendrogram of the example dataset exhibits high concordance (the measurements of Robinson–Foulds distances [21] were both equal to 0) between the test sets and our approach (Figure 2 and Figure 3).

Another real-case dataset that consisted of next-generation sequencing data for 34 *Salmonella* Typhimurium, sequenced by Leekitcharoenphon et al. [22], was also used to evaluate our approach. As illustrated in Supplementary Figure S1, the genetic relationships among the 34 isolates constructed using the cano-eMLST approach were highly concordant with the relationships of the isolates determined using the single nucleotide polymorphism (SNP)-based method (six foodborne disease outbreaks), as shown in a study by Leekitcharoenphon [22]. The detailed running times of jobs with different numbers of *Salmonella* isolates were compared with the default parameter setting on a desktop computer with two CPUs (6 cores, 12 threads, 2.10 GHz) and 64 GB RAM (Supplementary Figure S2).

## 4. Discussion

In our test, the schemes used for comparing the differences among isolates can dramatically reduce the number of loci from thousands to dozens. If the trees computed with the core genome scheme resemble those computed with the reduced core genome scheme, the reduced core genome loci are critically different from the compared isolates. Hence, the reduced core genome scheme may be used for typing, as well as in comparative genomic studies (e.g., for finding virulence factors). Currently, several popular genomic comparison tools, such as Mauve [23] and LAST [24], provide genome-to-genome comparison. The advantage of our tool is that it can be used to compare bunch isolates simultaneously and to extract the most different loci among the isolates compared for further studies.

This cano-eMLST program is expected to be available to clinical laboratories after an environment is established using the virtual machine we provided. The cano-eMLST can be used for rapidly capturing molecular information on the strains, such as the genetic relationship among strains and pathogenicity. Therefore, the program is highly useful, not only for investigating and controlling nosocomial infection in hospitals, but also for disease monitoring and epidemiological investigation. Because the clinical laboratories of hospitals are the target users of this tool, we have provided step-by-step documentation on the installation and use of this program (see https://sourceforge.net/projects/cano-emlst/files/).

The application of eMLST approaches for epidemiological surveillance is set to become a trend in the public health domain. Several schemes with various functions have been reported in the literature. In addition to typing, the analysis of the differences between compared strains may be crucial for identifying the pathogenesis or antimicrobial drug resistance factors. In this study, we describe a feature-selection approach that can select a small set of loci (usually dozens) and adequately reproduce the genetic relatedness construct typically based on a large set of loci (usually thousands). Moreover, this dramatic locus reduction and the simplicity of the tree-traversing approach could enable researchers to determine the differences between various isolates. Thus, the cano-eMLST approach may be a valuable method for undertaking comparative genomic studies.

## Figures and Tables

**Table 1:** The Loci Extractor pseudocode.

1	Import ETE toolkit and scikit-learn library
2	Read newick tree file to be traversal
3	**for** each node in tree:
4	**if** node is leaf:
5	continue
6	(subtree_1, subtree_2) = get_children (node)
7	**if** (leaf_amount(subtree_1) < 3 and leaf_amount(subtree_2) < 3):
8	continue
9	(class_1, class_2) = (subtree_1, subtree_2)
10	Informative loci selected by feature importance
11	Non-redundant merge of the informative loci

## Supplementary Materials

The following are available online at https://www.mdpi.com/2076-2607/7/4/98/s1.
