# Supplementary material for: The Cano-eMLST Program: An Approach for the Calculation of Canonical Extended Multi-Locus Sequence Typing, Making Comparison of Genetic Differences Among Bunches of Bacterial Strains
Source: Microorganisms. 2019 Apr 3;7(4):98. doi: 10.3390/microorganisms7040098 (PMC6517909; doi:10.3390/microorganisms7040098)
Supplement: Supplementary file 1 [file microorganisms-07-00098-s001.pdf]

The Cano-eMLST Program: An Approach for the Calculation of Canonical Extended Multi-Locus Sequence Typing, Making Comparison of Genetic Differences Among Bunches of Bacterial Strains

Yen-Yi Liu [1], Ji-Wei Lin [2] and Chih-Chieh Chen [2,3,4,*]

[1] Central Regional Laboratory, Center for Diagnostics and Vaccine Development, Centers for Disease Control, Taichung 40855, Taiwan; current788@gmail.com
[2] Institute of Medical Science and Technology, National Sun Yat-sen University, Kaohsiung 80424, Taiwan; jwlin@imst.nsysu.edu.tw
[3] Rapid Screening Research Center for Toxicology and Biomedicine, National Sun Yat-sen University, Kaohsiung 80424, Taiwan
[4] General Institute of Clinical Medicine, Kaohsiung Medical University, Kaohsiung 80708, Taiwan
* Correspondence: chieh@imst.nsysu.edu.tw; Tel.: +886-7-5252000 ext. 5791

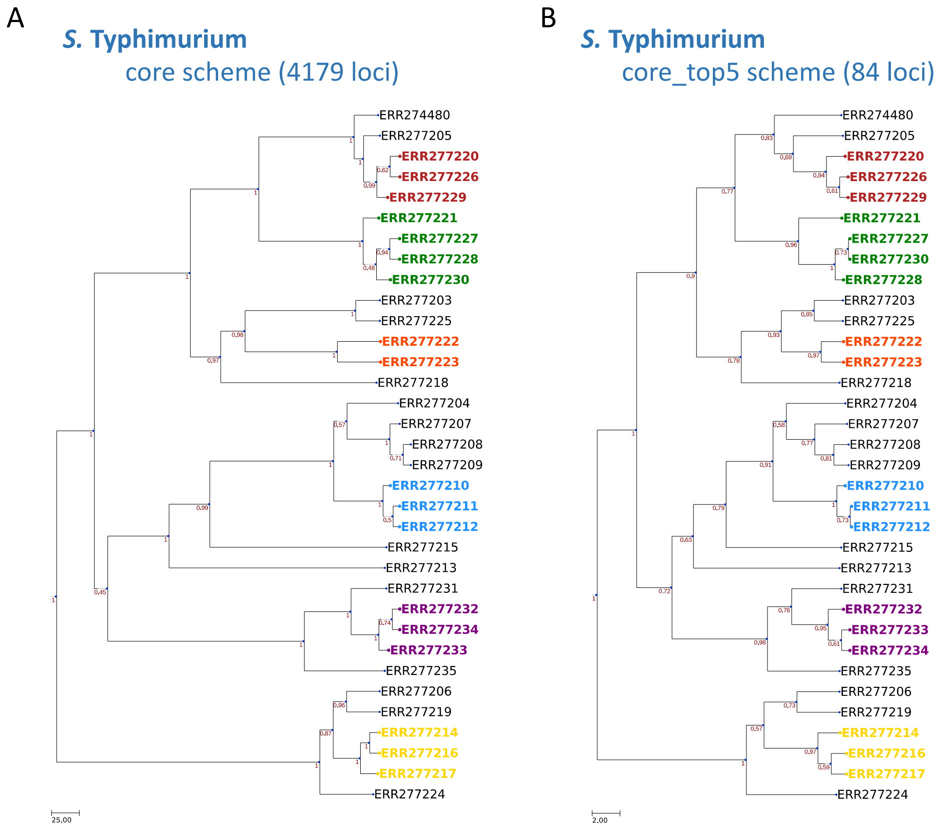

Figure S1. Dendrogram for 34 epidemiologically well-characterized *Salmonella* Typhimurium isolates sequenced by DTU Food [1]. The genetic relatedness trees were generated on the basis of (A) core scheme (4179 loci) and (B) core_top5 scheme (84 loci). Isolates of six foodborne disease outbreaks are colored in red, green, orange, blue, purple, and yellow, respectively. The core scheme refers to the core genome scheme generated in step 2 by the PGAdb Builder. The core_top5 scheme is a subset of the core scheme that unites the five most discriminatory loci for each split. The scheme was generated in step 5 by the Loci Extractor.

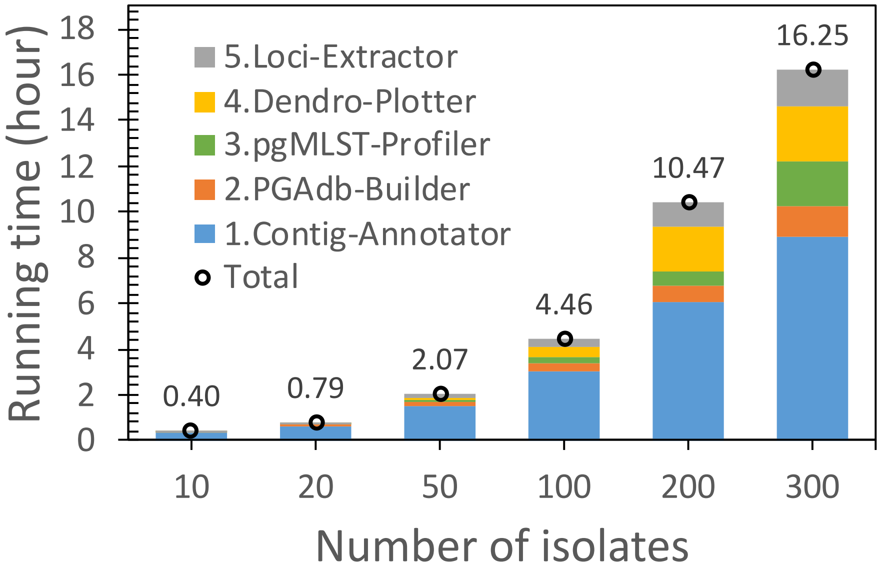

Figure S2. The running time versus the number of isolates. The detail running time comparison of jobs with different numbers of *Salmonella* isolates were tested with the default parameter setting on a desktop computer with two (6 cores, 12 threads, 2.10 GHz) CPUs and 64 GB RAM.

References

1. Leekitcharoenphon P, Nielsen EM, Kaas RS, Lund O, Aarestrup FM: Evaluation of whole genome sequencing for outbreak detection of Salmonella enterica. *PLoS One* **2014**, 9(2):e87991.
